# Supplementary material for: Listening to parents to understand their priorities for autism research
Source: PLoS One. 2020 Aug 13;15(8):e0237376. doi: 10.1371/journal.pone.0237376 (PMC7425861; doi:10.1371/journal.pone.0237376)
Supplement: S1 File — (DOCX) [file pone.0237376.s001.docx]

Sample Questionnaire

**Part one:** **Demographics**

*Please tell us about you and your family*

Note: If you have more than one child on the autism spectrum in the family, please base your answers to the following questions on only one of your children. If you wish, you can then complete the questionnaire again to tell us your priorities for your other child(ren).

1. Child age
2. Child gender
3. Number of children diagnosed with autism in the family

- One child
- Two children
- Three children
- Four children
- Five or more children

1. Parent age
2. Parent gender
3. Please select your highest level of education from the following:

- No formal education completed
- Completed secondary school
- Completed tertiary education

**Part two: Exploring research needs**

*We are interested to know what you as parents of children on the autism spectrum, want to see as the focus of autism research.*

1. In your opinion what three areas should research focus on to support your child on the autism spectrum in each of the following settings: Please list at least one or as many as three priorities for each setting using the free text boxes provided.

**Home School Community**

1.

2.

3.

1.

2.

3.
