# Supplementary material for: Listening to parents to understand their priorities for autism research
Source: PLoS One. 2020 Aug 13;15(8):e0237376. doi: 10.1371/journal.pone.0237376 (PMC7425861; doi:10.1371/journal.pone.0237376)
Supplement: Attachment — Submitted filename: PONE-D-20-01529 Reviewer Comments.docx [file pone.0237376.s002.docx]

Comments to the Author

Upon my review, I recommend the manuscript titled: “Listening to parents to understand their priorities for autism research” for a decision of Major Revision for publication in PLOS ONE. Overall, I find that this manuscript offers a strong contribution to the larger area of Autism research. It provides insight into the process of involving parent’s perspectives in research priority setting as well as an analysis of what some of these priorities are within the Autism community. I do however think that the arguments and organization of the manuscript need to be made stronger. Particularly, the overall justification and set up of the manuscript are unclear. While the results and discussion of the data are strong, I found myself coming to my own conclusion about the importance of this work, whereas the manuscript should have made this clearer from the start. I also think that the balance of a discussion of the process of involving parent’s perspectives and the actual data collected in for the manuscript should be considered further. I do find this work to be important to the field and important to share. I have provided some detailed comments below. The comments are in approximate chronological order in relation to the manuscript. I hope you find these comments helpful.

Reviewer Comments

-There are some inconsistencies in editing of the manuscript. For example, there seems to be both APA and MLA citations included in text on page 1, some paragraphs not being indented after headings, statements in the manuscript about redaction due to blind review, etc. Please review the entire manuscript for errors such as this.

-The introduction of the manuscript could benefit from a deeper discussion of why engagement is important.

-The discussion of the need for engagement with priority setting is not clear. It is stated that priority setting needs to be done and then two larger studies where it was done are outlined. There is also a point made about priority setting only being done for early intervention years, but then the studies outlined are not clearly described in terms of their support of this point. It seemed as though the other studies did measure more than just early intervention years. It would be helpful to clarify these points and connect the introduction arguments more directly to the findings of the reviewed studies.

-The introduction overall was weak in terms of how prior work was being connected to the current work. Is the reader supposed to see the current study as building on past works but with a small twist? Is the reader supposed to see the current study as replicating the past works?

-What is the Autism CRC? Please describe for the reader.

-Overall, the introduction but does not provide a strong argument for why this manuscript is being written and what the manuscript is trying to get across. It would be helpful to more directly explain to the reader what the point is.

-Why should another priority setting paper be conducted if two large-scale ones have already been done? Did the prior work miss something? Provide more justification in the introduction.

-I am not sure that I fully buy the argument that the research priorities of parents of school-aged children with autism is a gap in the literature. The manuscript described two studies in the introduction that explored research priorities. Was it supposed to be stated that those studies specifically did not evaluate parents of school-aged children? If this is the argument it needs to be made clearer or a new argument should be made regarding the importance of this work.

-I would like to see a brief justification as to why the 3 settings (home, school, community) were chosen.

-It would be helpful to have a brief explanation of the “forced sort” Q-sort task of Q-methodology within the manuscript.

-Nice description of the content analysis.

-The quotes from the families are very helpful in the discussion of why certain priorities ranked where they did during the Q-sort method.

-The parent discussion piece is very strong.

-Should there be any discussion about the location of the parents in terms of their responses? For example, are their responses different because they are all from Country X and the social systems in that country may be quite different from other countries, and therefore priorities may be different? Perhaps this is a point for discussion or limitations.

-It may be helpful to include a discussion on the broadness vs. narrowness of the identified priorities. Some seem to be quite broad like health and well being while others are very specific like meltdowns and tantrums. In making suggestions about future research on these priorities, it would be helpful to consider the scope and difficulty in addressing them.

-The discussion about the cognitive processes involved in the two methods is appreciated.

-I would like to see a more specific call to action in the discussion of the manuscript for research that is in the priority areas identified. The data from this study has clearly identified some gaps in the current research and those gaps should be highlighted in the current manuscript as a way to guide future work.

-The manuscript seems to err on the side of discussion of the importance of the process of gathering parents priorities and not as much on what was actually found in the study. There is importance to both and potentially more importance in the latter. I would encourage the authors to consider this balance in the overall presentation of the manuscript.
