# Supplementary material for: Listening to parents to understand their priorities for autism research
Source: PLoS One. 2020 Aug 13;15(8):e0237376. doi: 10.1371/journal.pone.0237376 (PMC7425861; doi:10.1371/journal.pone.0237376)
Supplement: Attachment — Submitted filename: PONE-D-20-01529_R1 Reviewer Comments.docx [file pone.0237376.s003.docx]

Comments to the Author – Revision 1

Upon my re-review, I recommend the manuscript titled: “Listening to parents to understand their priorities for autism research” for a decision of Accept for publication in PLOS ONE. I found the authors responses to my comments to be adequate and the manuscript to be better for it. Particularly, I find the arguments to be more well-rounded and the overall manuscript to have a much better balance of the discussion of the current study’s findings and the importance of the process of taking parents priorities into account. I think that what has been added to the manuscript has helped create a clearer description of the objectives of the manuscript and now provides a clearer call to action for readers. Overall this manuscript offers a great contribution to the field and I hope the authors are able to share their work here. Thank you for your continued work.
